# Enhancing patient safety in radiotherapy: Implementation of a customized electronic checklist for radiation therapists

**DOI:** 10.1016/j.tipsro.2024.100255

**Published:** 2024-05-28

**Authors:** Andrea Lastrucci, Marco Esposito, Eva Serventi, Livia Marrazzo, Giulio Francolini, Gabriele Simontacchi, Yannick Wandael, Angelo Barra, Stefania Pallotta, Renzo Ricci, Lorenzo Livi

**Affiliations:** a University of Florence, Florence, Italy; b Department of Allied Health Professions, Azienda Ospedaliero-Universitaria Careggi, 50134 Florence, Italy; c Medical Physics, The Abdus Salam International Centre for Theoretical Physics, Trieste 34151, Italy; d Radiation Oncology Unit, Santo Stefano Hospital, Department of Allied Health Professions, Azienda USL Toscana Centro, Prato 59100, Italy; e Department of Experimental and Clinical Biomedical Sciences “M. Serio” – University of Florence, Florence, Italy; f Medical Physics Unit – Careggi University Hospital, Florence, Italy; g Radiation Oncology Unit, Azienda Ospedaliero-Universitaria Careggi, 50134 Florence, Italy

**Keywords:** Radiotherapy, RTTs, Checklist, Risk management

## Abstract

**Introduction:**

The radiotherapy workflow involves the collaboration of multiple professionals and the execution of several steps to results in an effective treatment. In this study, we described the clinical implementation of an electronic checklist, developed to standardize the process of the chart review prior to the first treatment fraction by the radiation therapists (RTTs).

**Materials and Methods:**

A customized electronic checklist was developed based on the recommendations of American Association of Physicists in Medicine (AAPM) Task Groups 275 and 315 and integrated into the Record and Verify System (RVS). The checklist consisted of 16 items requiring binary (yes/no) responses, with mandatory completion and review by RTTs prior to treatment. The utility of the checklist and its impact on workflow were assessed by analysing checklist reports, and by soliciting feedback to RTTs through an anonymized survey.

**Results:**

During the first trial phase, from June to November 2023, 285 checklists were completed with a 98% compilation rate and 94.4% review rate. Forty errors were detected, mainly due to missing signed treatment plans and absence of Beam’s Eye View documentation. Ninety percent of detected errors were fixed before the treatment start. In 4 cases, the problem could not be fixed before the first fraction, resulting in a suboptimal first treatment. The feedback survey showed that RTTs described the checklist as useful, with minimal impact on workload, and supported its implementation.

**Discussion:**

The introduction of a customized electronic checklist improved the detection and correction of errors, thereby enhancing patient safety. The positive response from RTTs and the minimal impact on workflow underscore the value of the checklist as standard practice in radiotherapy departments.

## Highlights

• This study investigates the impact of implementation of a customized electronic checklist, developed to standardize the process of the chart review prior to the first treatment fraction.
• The analysis of the data highlighted the mistakes identified by Radiation Therapists while compiling checklists.
• The use of a checklist plays a crucial role in improving detection and correcting errors.

## Introduction

Radiotherapy is a complex treatment modality that uses ionising radiation to treat, palliate and cure cancer patients[1]. The radiotherapy process involves the synergic work of multiple professionals and develops in several steps: planning CT acquisition, targets and organs at risk delineation, optimization of a treatment plan and treatment delivery[1], [2], [3]. To ensure safe and successful radiotherapy, the treatment accuracy must be guaranteed: it is crucial that the correct dose is delivered to the planned target volume of the right patient[3]. Thus, careful assessment and review of the initial patient treatment chart, prior to the first dose delivery, is necessary to minimise risk of errors throughout all workflow stages [4]. Careful chart review is an effective proactive safety method for identifying and addressing potential errors before treatment, making it a critical component of ensuring safety[5], [6].

Radiation Therapists (RTTs) play a major role in patient positioning, ccording to the setup instructions and treatment plan, and also in ensuring safe treatment delivery[4], [7], [8]. Indeed, the scientific literature reports the key role of RTTs in the initial chart review, checking various geometric and dosimetric parameters, as well as the presence and completeness of key clinical documents[1], [4], [8]. The American Association of Physicists in Medicine (AAPM) Task Group 275 made practical and evidence-based recommendations on how to perform a chart review for radiation treatments[5]. Subsequently, AAPM Task Group 315 made specific recommendations for the initial review of the plan and for items to be checked[4]. The checklist is one of the tools proposed in the two AAPM reports for carrying out check controls[4], [5]. This safety tool provides a useful memory guide to ensure that all items checked during the chart review are not overlooked, enabling healthcare professionals to concentrate on the complex tasks that demand their full attention[9], [10]. According to the literature, checklists have been proven to standardize practices, enhance the ability to identify and prevent errors and improve patient workflow[2], [11], [12], [13].

In this study, we described the clinical implementation of an electronic checklist, developed to standardise the RTT chart review prior to the first treatment fraction, and analysed the results obtained in the trial phase.

In clinical practice, before the implementation of the electronic checklist, RTTs conducted treatment chart reviews. However, several issues had emerged concerning different aspects. These issues included ensuring the completion of all required checks, handling the resolution of warnings, and the absence of a standardized and structured process for conducting the necessary checks. In addition, we conducted an anonymous survey among RTTs to explore their perceptions of the daily use of the checklist and their level of satisfaction. While the development and use of checklists in radiotherapy have recently increased, there is a lack of evidence in the literature exploring the implementation of a digital pre-treatment checklist developed following the data reported in AAPM task groups 275 and 315. The present trial was conducted to provide prospective data on this crucial clinical issue, testing the real-world impact of this strategy to minimize the risk of errors in routine practice.

## Methods and Materials

### Checklist development

**Fig. 1:**
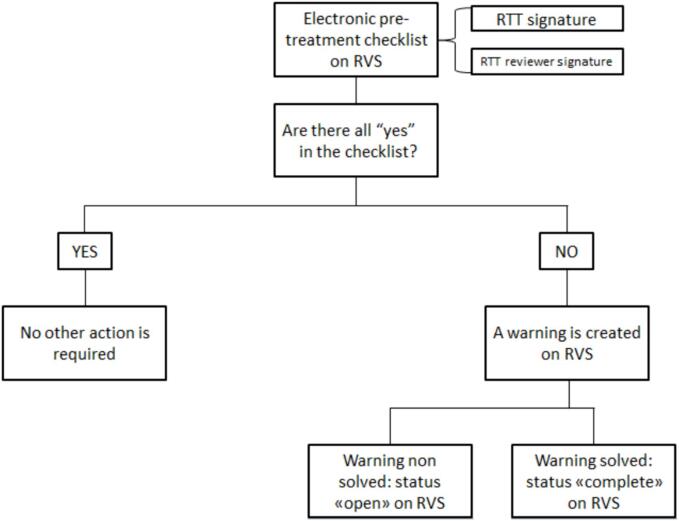
Workflow for completing electronic pre-treatment checklist.

A customized electronic checklist for initial chart review was developed by 2 RTTs following the recommendations of AAPM task groups 275 and 315[4], [5]. Specifically, for the selection of items, the results of risk analysis methods using Failure Mode and Effects from AAPM 275 were analysed, and an integration and evaluation of the example checklist from AAPM 275 and 315 were made to build our own. The electronic checklist was reviewed by all RTTs to verify its clarity before proceeding. The final electronic checklist consisted in 16 items and is summarized in Table 1. As suggested in the literature[1], [14], the questions in the checklist were formulated in a simple, direct mode and assuming only binary responses (yes/no). The electronic checklist was integrated into the Record and Verify System (RVS) (Mosaiq v2.64, Elekta Medical Systems). It was designed to be compiled by two RTTs the day before each new radiotherapy treatment. At the end of the compilation, two electronic signatures were required using the RVS credentials: the first representing the operator compiling the checklist, and the second representing the verifier of the compiled checklist. Compilation of all items was mandatory, and if one or more items were forgotten, the checklist was incomplete and therefore could not be signed. If the reviewer RTT detected discrepancies in the completed checklist, he could amend it. Once signed, the checklist remains on file and can be accessed by all radiotherapy staff. If all items reported no error, no warning was issued. However, if one or more items were answered with “no”, indicating errors or omissions in the planning or simulation phase, a special warning was generated in the RVS. The status of the warning could be viewed and edited. When the warning was resolved, a full status update was uploaded by the RTT. For each activated warning, there was a corresponding procedure for problem resolution, which involved alerting the physician and physicist responsible for the treatment. All created warnings were recorded in a database for analysis, and for developing specific improvement actions. Fig. 1 illustrates the activities carried out when during checklist completion.

**Table 1:** Electronic pre-treatment checklist items.

Items	Yes	No
Are patient name and identification data correct? Cross-check between RVS and chart.		
Is patient consent form signed by RO?		
Is paper treatment plan signed by RO and physicist?		
Are treatment site and plan laterality correct? Cross-check between RVS and chart.		
Are prescription and treatment fields approved by RO and Physicist respectively?		
Are Tx technique, number of fractions, total dose and dose/fraction correct? Cross-check between RVS and chart.		
Is site patient set-up (which includes information about immobilization device set-up, positioning, and skin markers) on RVS compiled and signed by RTT?		
Are SSD parameters present on RVS and paper plan?		
Are the geometric parameters of the field correct? Cross-check between RVS and chart.		
Is Reference CT sent to third party system and is a right acquisition protocol set?		
Is BEV present on RVS and paper plan? (only for 3D-CRT Tx technique)		
Are there information about patient’s preparation on RVS or paper chart?		
Is patient’s surface uploaded correctly on SGRT software? (only for breast cancer patients)		
Is third party IGRT data ready? Check corrected images input to third party image systems.		
Has the treatment been scheduled correctly on RVS? Check number of fractions and optimization of sequencing fields scheduled.		
Are Tx appointments correct? Check on RVS the presence of conflicts.		
*RTT signature*		
*RTT reviewer signature*		

BEV: Beam's eye view; IGRT: Image Guided Radiotherapy; RTT: Radiation Therapist; RO: Radiation Oncologist; RVS: Record and Verify System; SGRT: Surface Guided Radiotherapy; SSD: Surface Skin Distance; Tx: treatment; 3D-CRT: three-dimensional conformal radiation therapy.

As suggested in literature[15], [16], [17], blended learning was preferred to the traditional approach and was chosen for training RTTs to complete the electronic pre-treatment checklist. The training period lasted approximately 40 days, and during the first month of trial phase, a weekly briefing was organised among the RTTs to discuss and resolve any issues related to the compilation of the checklist that had arisen during the previous week. To assess the RTTs' participation in completing the checklists, the compilation/coverage rate, defined as the ratio of completed checklists to the total number of checklists to be completed, and the review rate, which is the ratio of reviewed checklists to the total number of checklists to be reviewed, were analysed.

### Survey

An invitation to complete an anonymous online questionnaire was sent via email at the end of the study phase to all RTTs involved in completing the checklist (n = 7). The questionnaire contained a series of 10 questions that required either one dichotomous or three responses and asked participants to choose between agreeing or disagreeing. The questionnaire is included in the supplementary material.

## Results

### Data Analysis Checklist

During the first trial phase, which lasted 6 months from June to November 2023, a total of 285 checklists were completed. The compilation/coverage rate was 98.6 %. Of these completed checklists, 269 checklists were reviewed and signed by second RTTs, representing a review rate of 94.4 %. In terms of treatment techniques, out of the 285 completed checklists, 65.3 % (n = 186) referred to three-dimensional conformal radiation therapy (3D-CRT), 34.0 % (n = 97) to volumetric modulated arc therapy (VMAT) and the remaining 0.7 % (n = 2) to dynamic multileaf collimator (DMLC) treatments.

**Fig. 2:**
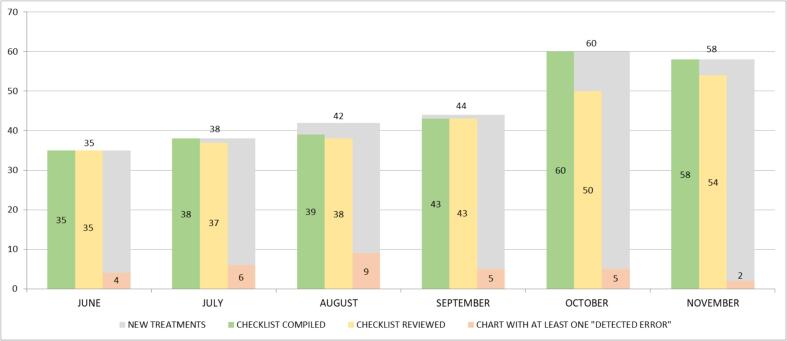
Monthly distribution of the number of initial treatments, checklists compiled, checklists reviewed and checklists with at least one “warning created”.

A total of 32 completed checklists reported at least one or more “detected errors” (11.2 %), and overall, 40 warnings were created during this period. The histogram in Fig. 2 illustrates the monthly distribution of compiled checklist, reviewed checklists, and those rated with at least one “detected error“, in comparison to the number of initial treatments.

**Fig. 3:**
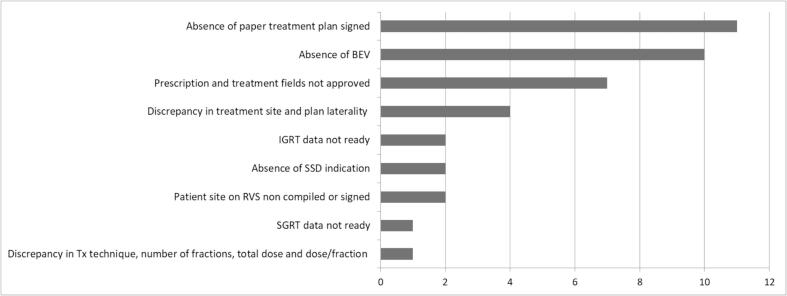
Distribution of the warning types during the trial phase.

The distribution of the warning types created is illustrated in Fig. 3. The majority of alert found during the initial chart review were the absence of the paper treatment plan signed by the RO and physicist (n = 11), followed by the absence of Beam’s eye view (BEV) (n = 10). More than 80 % of the warnings created were solved and changed status on RVS before the first treatment (82.5 %, n = 33). Upon analysing the treatment chart and RVS for the 7 unresolved warnings, it was found that 3 of them were resolved, but the RTTs did not change the status of the warning in the RVS. All 4 unresolved warnings concerned the absence of BEVs on the chart and in the RVS.

**Fig. 4:**
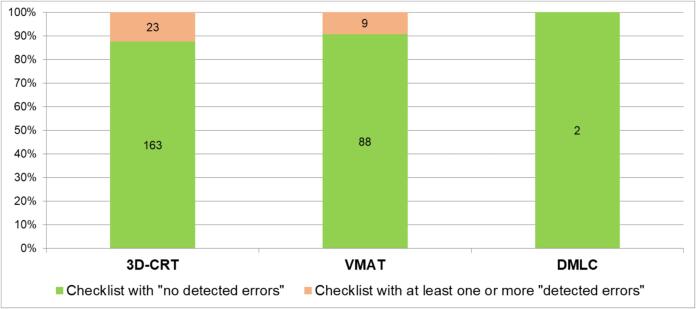
Percentage distribution of the information on the treatment techniques on the checklists with a distinction between checklists with “no detected errors” and at least one or more “detected errors” for each treatment technique.

The majority of the checklists with at least one or more “detected errors” (72 %, n = 23) were related to 3D-CRT plans, while the remaining 28 % (n = 9) were related to VMAT plans. The histogram in Fig. 4 summarises graphically the data on treatment techniques in

the checklists.

### Data analysis survey

All RTTs completed the online survey. The majority of respondents have more than 15 years of radiotherapy experience (n = 3), while the remaining RTTs have less than 4 years of experience (n = 2) and between 4 and 10 years of experience (n = 2), respectively. All RTTs agreed that the electronic pre-treatment checklist was useful and enabled them to improve safety during the initial chart review. Most RTTs (5 out of 7) found that completing the checklist was not an excessive workload in daily practice. Additionally, all RTTs felt that the checklist items were appropriate for conducting the initial chart review. They did not suggest any modifications and expressed their recommendation for the implementation and use of this electronic checklist in other radiotherapy departments. The questionnaire also revealed that 42.8 % of responders (n = 3) encountered problems related to managing possible errors on the RVS resulting from the checklist, but not with visualisation. Conversely, the same number felt that both the management and visualisation of possible errors on the RVS are simple and intuitive. Only one respondent stated that both the management and visualisation are complex. All responders agreed that the teaching and organisational methods covered in the training were appropriate regarding the content to be covered.

The majority of responders (n = 4) indicated that the time taken to compile the checklist was between 6 and 10 min. For 2 RTTs, the completion time was less than 5 min, and only one indicated between 11 and 15 min. Table 2 summarizes the data obtained from the compilation of the questionnaire.

**Table 2:** Results obtained from the compilation of the questionnaire.

**Questions**	**Answers**	**N**
Radiotherapy experience in years	<4years	2
	4–10 years	2
	>15 years	3
Usefulness of electronic pre-treatment checklist	Yes	7
	No	0
Electronic pre-treatment checklist improves safety	Yes	7
	No	0
Modifications required for the checklist	Yes	0
	No	7
Checklist compilation causes an excessive workload	Yes	2
	No	5
Checklist items are appropriate for conducting the initial chart review	Yes	7
	No	0
Time requested for checklist compilation	1–5 min	2
	6–10 min	4
	11–15 min	1
Export and share this checklist with other radiotherapy departments.	Yes	7
	No	0
The visualization and management of resolving warning are simple and intuitive	Yes	3
	Only visualization is simple	3
	No	1
The teaching and organisational methods covered in the training were appropriate	Yes	7
	No	0

## Discussion

Our study involved the clinical implementation of an electronic checklist aimed at standardizing the chart review process before the first treatment fraction. The checklist was developed based on the recommendations of AAPM Task Groups 275 and 315 and integrated into the RVS. The results indicate that the checklist was completed by at least one RTT in 98 % of new treatments and reviewed and signed by a second RTT in 94 %, demonstrating minimal impact on workflow. However, feedback from the questionnaire revealed a limited impact on RTTs' workload: one RTT reported taking between 10 and 15 min to complete the checklist, while 4 RTTs reported taking between 5 and 10 min. Two out of seven RTTs reported that this caused an excessive workload. This suggests that the 16 items to be completed may represent an appropriate compromise between exhaustiveness and workload.

Regarding the management of resolving warnings, responses highlighted the challenges faced by RTTs. Subsequent to these responses, a briefing was conducted to address the identified difficulties and suggest improvement measures. The primary challenges in addressing alerts were associated with the workflow within the R&V system, necessitating intervention in the “Quality Checklist” section for resolution. To address this, specific visualization templates and search criteria were incorporated into this section, and additional training on the R&V system was provided to RTTs. Although the number of checklists to be completed increased by 65 % from the first to the last month, the completion and review rates remained stable, confirming that the compilation of checklists was feasible with little additional effort. We can make some interesting observations about the errors found by using the checklist:

More than 90 % of the errors were corrected before the start of treatment, leading to a proactive effect of reducing errors of varying severity. The most noticeable errors were attributed to staff forgetfulness. However, not all errors found could be corrected. In four cases, the imaging data from (BEV) were not available, and treatment commenced without it, resulting in a suboptimal condition. To solve this situation, it was suggested to have a multidisciplinary discussion with all professionals involved (e.g. physicists, physicians and RTTs) about the errors found and the necessary procedural improvements to further limit their presence.

Following the multidisciplinary discussion, changes were made to the practices of Radiation Oncologists and Physicists. In particular, as suggested in the literature[18], [19], [20], an independent double-checking procedure was introduced in clinical practice by the Radiation Oncologists and Physicists to improve error detection and ensure safer treatment delivery. This double check takes place after − treatment planning preparation and is carried out for every new treatment, regardless of the treatment technique.

The majority of the checklists with “detected errors“ items were related to 3D-CRT treatments. This can be attributed to the fact that many 3D-CRT treatments were performed in an acute medical setting (e.g. spinal cord compression) where it is important to minimise unnecessary delays in treatment that could compromise a potential curative outcome[21]. Under these circumstances, it is possible that some information may be forgotten or omitted during the radiotherapy pathway before treatment.

Our experience is consistent with a few other papers found in the literature[2], [22], [23], [24], [25], [26], [27]. There were only three studies in the literature on the use of checklists in the pre-treatment or pre-start phase, as recommended by AAPM Working Groups 275 and 315, which were conducted by RTTs [1], [8]. In particular, in the study of Kalapurakal et al., conducted in USA from 2001 to 2011, the use of checklists and timeouts for all RT staff was implemented, and RTTs were involved in the compilation of several checklists during entire RT process. By implementing these proactive measures, various types of errors were significantly reduced, and mistakes involving incorrect patients, sites, and doses were completely eliminated.

In the study of Mukundan et al., conducted in India in 2021[1], a checklist was developed to reduce the possibility of errors before and during treatment delivery on a telecobalt machine, while in the study of Younge et al., conducted in the USA in 2017[8], a therapist pre-start QA checklist was developed and completed by RTTs.

According to literature[1], [4], the checklist was a useful tool for standardising and streamlining the RTTs' workflow during check and review the patient charts before starting treatments. The electronic checklist was compiled by two RTTs and stored on the RVS system. This allows for a retrospective analysis of the completed checklists and enables a multidisciplinary examination of the most common errors, facilitating the implementation of improvement actions.

After this trial phase, the checklist for the initial review of the patient record was then introduced into clinical practice. As a future development of this work, it will be interesting to analyse the results of the multidisciplinary review discussions with all healthcare professionals involved in the RT process.

While the results of the trial phase were analysed in this study, future analysis will be necessary to assess the impact of improvement actions on the reduction of errors by analysing future checklists.

## Conclusion

In this work, a customized electronic checklist was developed to facilitate error detection prior to treatment initiation and implemented clinically with minimal impact on the normal workflow of RTTs. Our results confirm that the use of a customized checklist in the chart review effectively detects and corrects several errors prior to the commencement of treatment, thereby improving patient safety. Building on the results of this study, we have introduced a multidisciplinary meeting dedicated to discussing the errors identified by the checklist.

## Declaration of competing interest

The authors declare that they have no known competing financial interests or personal relationships that could have appeared to influence the work reported in this paper.
