# Supplementary material for: Enhancing patient safety in radiotherapy: Implementation of a customized electronic checklist for radiation therapists
Source: Tech Innov Patient Support Radiat Oncol. 2024 May 28;31:100255. doi: 10.1016/j.tipsro.2024.100255 (PMC11176772; doi:10.1016/j.tipsro.2024.100255)
Supplement: Supplementary Data 1 [file mmc1.docx]

**SUPPLEMENTARY MATERIAL**

| **Questions** | **Answers** |
| --- | --- |
| 1. What is your experience in radiotherapy, in years? | <4 years |
|  | 4 -10 years |
|  | 11 –15 years |
|  | >15 years |
| 1. Do you find the electronic pre-treatment checklist useful? | Yes |
|  | No |
| 1. Do you Electronic pre-treatment checklist improve your safety? | Yes |
|  | No |
| 1. Would you like to make any modifications to the checklist? | Yes |
|  | No |
| 1. Does compiling the checklist cause an excessive workload? | Yes |
|  | No |
| 1. Are the checklist items appropriate for conducting the initial chart review? | Yes |
|  | No |
| 1. What is the amount of time requested for checklist compilation? | 1-5 minutes |
|  | 6-10 minutes |
|  | 11-15 minutes |
|  | >15 minutes |
| 1. Would you recommend exporting and advising this checklist to other Radiotherapy departments? | Yes |
|  | No |
| 1. Is the visualization and management of solving warnings simple and intuitive? | Yes |
|  | Only visualization is simple |
|  | No |
| 1. Were the teaching and organizational methods covered in the training appropriate? | Yes |
|  | No |

Survey Questionnaire: Comprehensive Question List
